# MambaAdapt: Joint Descriptor–Alignment Learning with Mamba–Transformer Fusion for Robust Long-Term VPR

**DOI:** 10.3390/s26185799

**Published:** 2026-09-13

**Authors:** Muhammad Fahad, Di He, Wenxian Yu, Trieu-Kien Truong

**Affiliations:** 1 School of Integrated Circuits, Shanghai Jiao Tong University, Shanghai 200240, China; engineerfahad2010@alumni.sjtu.edu.cn (M.F.); truong@isu.edu.tw (T.-K.T.); 2 School of Automation and Intelligent Sensing, Shanghai Jiao Tong University, Shanghai 200240, China; wxyu@sjtu.edu.cn; 3 Shanghai Key Laboratory of Navigation and Location-Based Services, Shanghai Jiao Tong University, Shanghai 200240, China

**Keywords:** MambaAdapt, visual place recognition, long-term autonomous navigation, Mamba–Transformer fusion, CT-MRRE, cross-temporal alignment, adaptive feature reweighting, continual learning

## Abstract

Visual place recognition (VPR) is one of the important enabling technologies in localization and mapping for intelligent transportation systems (ITSs). Long-term VPR is challenging even with descriptors extracted exclusively for the discrimination of position, especially when there is a significant change in illumination conditions, seasons, viewpoints, occlusions, traffic, and/or changes in structures. Additionally, temporal adaptation is typically done after the learning of the descriptors, and adaptation goals do not directly impact the descriptor space, which can act as a conflict with existing place representations. In order to overcome this limitation, the MT-FusionNet descriptor backbone is jointly optimized with the Cross-Temporal Meta-Reweighting Recurrent Encoder (CT-MRRE) and a representation-preserving process with replay. The two steps of CT-MRRE are: residual cross-temporal alignment to correct the descriptors dislocated by the condition and suppressing unreliable descriptors in the context of the current environmental condition through recurrent reliability reweighting. Therefore, place discrimination, temporal adaptation, reliability estimation, and representation retention are all optimized in a single descriptor space. The average Recall@1 is 97.2% across the four sites, being 96.4%, 96.2%, 99.1%, and 97.0% for Oxford RobotCar, Nordland, City Center, and St Lucia, respectively. This is in line with the common four-dataset evaluation protocol, where it represents an average absolute gain of 2.8 percentage points over MT-FusionNet and of 3.7 percentage points over CerfeVPR. The complementary effect of the cross-temporal alignment, context-aware reliability reweighting and replay-supported joint optimization is revealed by the component analysis. These results show the usefulness of the combination of discriminative descriptor learning, cross-time adaptation, reliability weighting, and representation preservation in the evaluated environmental variations for long-term VPR.

## 1. Introduction

Visual place recognition (VPR) is an essential capability for localization and autonomous navigation in intelligent transportation systems (ITSs), as it estimates a vehicle’s location by matching the current sensory observation with previously learned places [1]. When there are significant variations in lighting, weather, season, focus, traffic, occlusion, or scene structure, correct recognition becomes challenging. The problems with global descriptors (NetVLAD [2], CosPlace [3], MixVPR [4], and EigenPlaces [5]) are that they tend to have a fixed image-to-descriptor mapping but do provide improved discrimination and compactness. These sorts of static measurements may still be susceptible to drift over time and environmental conditions, which are not modeled at the training stage [6,7].

There are two current directions in VPR research, each complementary to the other. Descriptor-centric work enhances spatial and long-range representation learning by employing convolutional networks, Transformers, and selective state-space models [8]. Adaptation-oriented work performs spatio-temporal alignment [9], sequence matching [10,11], or implements continual learning mechanisms [12,13] to achieve the desired effect. These components can be trained following a fixed descriptor. If a descriptor is frozen, it can no longer be reshaped by downstream alignment and reliability goals, and future updates may be used to overwrite place representations that may be useful.

In this paper, we build on the MT-FusionNet descriptor backbone we had previously developed [14] to create a unified long-term VPR framework, MambaAdapt. The novelty of this study lies not in the CNN–Mamba–Transformer fusion but in jointly adapting the learned descriptor space using the proposed CT-MRRE module and replay-supported representation preservation. CT-MRRE unites residual cross-temporal alignment with a recurrent version of context-aware reliability re-weighting; the joint objective enables discrimination, adaptation, reliability, and retention objectives to jointly influence the descriptor backbone throughout training.

The primary research question concerns the possibility of optimizing a hybrid visual descriptor together with the temporal alignment, context-aware estimation of the reliability of the features, and the preservation of the representation. Therefore, MambaAdapt learns a shared representation space which enables place discrimination, temporal correction, reliability weighting and resistance to catastrophic forgetting.

Furthermore, MambaAdapt is tested on the Oxford RobotCar [15], Nordland [16], City Centre, and St Lucia [17]. These datasets include day–night illumination variation, seasonal appearance change, urban viewpoint variation, occlusion, repetitive structure, and time-of-day change. Related descriptor-learning and adaptation methods are briefly summarized in Section 2. Section 3 introduces MambaAdapt; Section 4 introduces the experimental protocol; Section 5 presents comparative and component analyses; Section 6 discusses limitations; and Section 7 summarizes and concludes the paper.

### 1.1. Problem Statement

While significant advances have been made in visual descriptor learning, long-term visual place recognition remains challenging when illumination changes, seasonal variation, viewpoint changes, occlusion, dynamic objects, and structural changes occur simultaneously. Most existing approaches focus primarily on optimizing visual descriptors for place discrimination, while treating temporal correction, feature reliability evaluation, and continual adaptation as separate stages. This separation prevents adaptation objectives from shaping the descriptor space itself, allowing condition-dependent features (e.g., illumination, vegetation, shadows, and transient objects) to dominate similarity estimation. Moreover, continuous adaptation without memory mechanisms can overwrite previously learned place representations and lead to catastrophic forgetting.

The research problem addressed in this study is therefore to design a unified VPR framework that jointly learns discriminative visual descriptors, nonlinear cross-temporal alignment, context-aware feature reliability, and representation preservation. To address this problem, MambaAdapt integrates the MT-FusionNet descriptor backbone, the CT-MRRE adaptation block, and replay-supported learning into a single end-to-end optimization framework.

### 1.2. Key Contributions

This work contributes the following:

- Unified MambaAdapt architecture: We propose MambaAdapt as a unified end-to-end framework for robust long-term visual place recognition. It combines the hybrid CNN–Mamba–Transformer descriptor of MT-FusionNet and CT-MRRE, an internal adaptation block for cross-temporal alignment and recurrent reliability reweighting.
- Joint descriptor–adaptation optimization: MambaAdapt jointly optimizes the goals of discrimination, alignment, reliability, and stability, together with the goal of replay consistency, through the descriptor backbone and the adaptation block. This allows the descriptor space to become more resilient to environmental variations for temporal alignment.
- Temporal robustness in context: CT-MRRE learns the residual transformation between reference and query conditions and produces a context-dependent reliability mask that suppresses dimensions that are unreliable in the query context while maintaining reliable structure information.
- Representation preservation with replay: Training with a stratified experience-replay mechanism keeps samples from different time steps during training and prevents representation changes that would destroy earlier learned place representations.
- Comprehensive experimental evaluation: MambaAdapt is compared with state-of-the-art and recent VPR techniques through incremental component analysis, environmental robustness evaluation, computational analysis, and qualitative error analysis on four widely used public long-term VPR benchmarks.

## 2. Related Works

VPR methods can be roughly classified into two approaches: descriptor-centric and adaptation-oriented approaches. Descriptor-centric approaches are associated with learning discriminative visual representations [18], while adaptation-oriented approaches are linked with coping with changes in the environment over time using temporal reasoning and feature correction, reference-set updating, and continual learning. Such directions have been separately generated, either as powerful static descriptors or as adaptive modules operating upon fixed representations. In previous work [14], we proposed a hybrid descriptor framework called MT-FusionNet. This is expanded in the current study by the joint optimization of the proposed CT-MRRE module and MT-FusionNet descriptor, which is called MambaAdapt. Thus, this work does not introduce CT-MRRE or any of its branches as separate methods but rather the complete adaptive VPR architecture.

### 2.1. Global Descriptor Learning for VPR

In VPR, global descriptor learning is a fundamental research area, as it facilitates concise and efficient scene representation. NetVLAD [2] formulated end-to-end trainable VLAD pooling with convolutional features, which was widely adopted as a basic method. More recent approaches enhanced the scalability and discrimination of descriptors. CosPlace [3] cast place recognition as a classification task to learn global representations that are geographically diverse. MixVPR [4] devised feature-mixing operations on global descriptors to reduce their size, and EigenPlaces [5] used eigenvector-based clustering while training to improve viewpoint resistance. Efficiency and robustness are also explored in recent works on pruning and ternary quantization [19], rotation-invariant aerial representations [20], teacher–student knowledge distillation [21], and dynamic feature enhancement with dual-path architectures [22]. However, these remain simplified, static, image-based descriptors, regardless of environmental changes or travel duration. These are restricted to long-term appearance variations due to changes in light, weather, viewpoint, and season [6,7]. Recent studies have explored illumination-robust global descriptors by incorporating frequency-domain representations and whitening strategies to improve feature stability under challenging lighting conditions [23].

### 2.2. Hybrid CNN, Transformer, and State-Space Descriptors

Hybrid architectures using complementary architecture components have been explored in recent VPR research. Self-attention-based global feature learning has been introduced in Vision Transformers (ViTs) [24], which allow long-range modeling. Later approaches included the cross-spatial pyramid attention [25], contrastive pair-classification learning in ViT for place recognition [11], and ternary Transformer-based compact representations for efficient VPR [12]. In recent years, selective state-space models like Mamba have introduced efficient sequential modeling capabilities. VMamba [13] applied Mamba to extend Mamba architectures for vision tasks and achieved near-linear complexity for both spatial and temporal representation learning. The advancements highlight how CNNs, Transformers, and Mamba accomplish complementary information: CNNs develop neighborhood structures, Transformers model global dependencies, and Mamba captures sequential dependencies. In our previous MT-FusionNet [14], we used a ResNet backbone [8] and a Transformer encoder to create a 512-dimensional descriptor. In contrast to regular frozen descriptors, MambaAdapt optimizes the descriptor together with the suggested adaptation objective.

Recent work also demonstrates that benefits of representation learning with Mamba can be extended beyond architectural fusion. In the case of multi-exposure HDR reconstruction, the complementary frequency information is separated using the frequency-decoupled state-space modeling (FD-HDRMamba) [26], while specialized Mamba experts are used to boost representation learning across domains (Sparse Mixture of Mambas) [27]. In addition to Mamba-specific architectures, coarse-to-fine Adaptive Alignment Representation (AAR) [28] shows that selective adaptive alignment and reweighting can be used to suppress nuisance-dominated information when misaligned and occluded, and BGA [29] illustrates the more general application of adaptive gating for noise-robust representation learning. In these studies, task-specific adaptive representation mechanisms emerge, but MambaAdapt is developed for long-term VPR through co-learning of cross-temporal descriptor alignment, context-dependent reliability reweighting, and replay-representational preservation.

### 2.3. Temporal Adaptation and Continual Learning in VPR

Another research area is long-term adaptation of VPR systems to a changing environment. In spatio-temporal alignment methods, the information about the sequences is utilized to align the descriptors [30], and in sequence-to-frame matching approaches, the adjacent frames are utilized for stable recognition with gradual changes [9,10]. Other approaches modify the retrieval databases using reference set modification and map-density approaches [7]. However, continuous deployment also comes with its own problem: catastrophic forgetting—that is, the loss of previous knowledge when adapting to new environments. For continual learning and incremental place recognition, solutions like experience replay [31] and elastic weight consolidation (EWC) [32] have been investigated. In the MambaAdapt model, the notions of adaptation are applied in the training process and embedded in the CT-MRRE. Its branch over time is based on neural-field deformation for descriptor alignment, and its recursive meta-reweighting branch is based on a meta-learner [33] to learn descriptor reliability weights from temporal and environment information. Studies on adaptive weighting and enhancement across different environments [34,35,36] confirm the significance of context-specific correction. But previous approaches mostly focus on adapting after descriptor learning rather than joint optimization.

### 2.4. Positioning of the Present Work

Further research at VPR has investigated related topics including, but not limited to, improved global descriptor learning [3,4,5,11,12,14,23,35], spatio-temporal and sequence matching [9,10,30,37], adaptation at test time or through a reference set [7], and continual learning mechanisms [31,32,34]. While these techniques increase either visual discrimination or adaptation to changing environmental conditions, the corresponding visual goals are usually optimized separately. The distinguishing contribution of MambaAdapt is not the CNN–Transformer–Mamba fusion itself, which was introduced in [14], but the cross-temporal adaptation and reliability modeling mechanisms developed on top of this descriptor backbone. To this end, the present work simultaneously optimizes this backbone for descriptors, alongside the proposed CT-MRRE module, which incorporates residual cross-temporal descriptor alignment, recurrent context-dependent descriptor reliability reweighting in a descriptor shared space, and replay-based descriptor representation preservation. Thus, place discrimination, time correction, estimation of reliability, and representation retention are jointly integrated into the learned representation rather than being treated as separate post-processing stages.

## 3. Proposed MambaAdapt Framework

**Figure 1:**
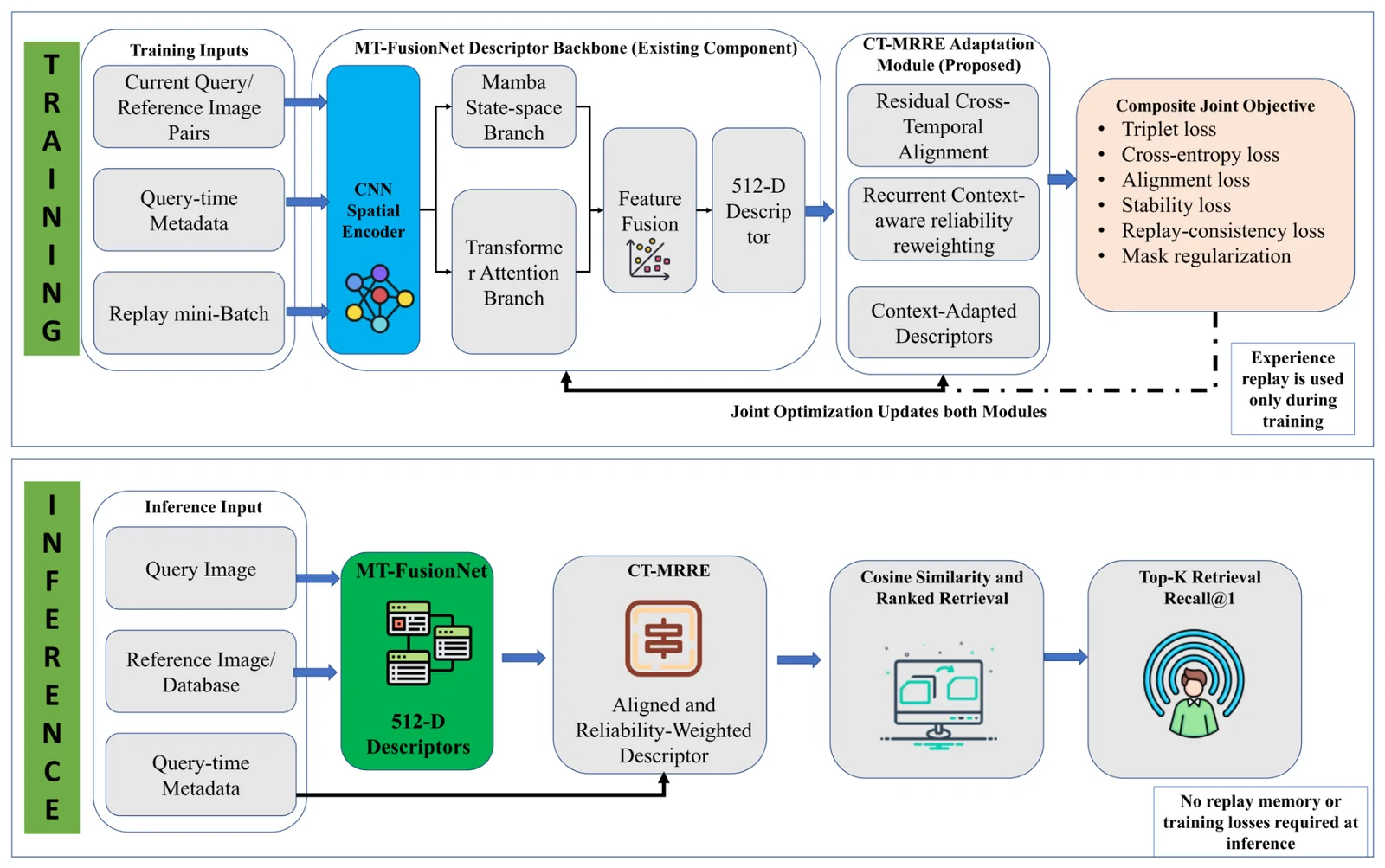
Conceptual overview of the proposed MambaAdapt framework. The proposed CT-MRRE aims to achieve context-conditioned residual cross-temporal alignment and recurrent reliability reweighting. In training, the descriptor backbone and CT-MRRE are updated simultaneously using a combination of the discrimination, alignment, stability, replay-consistency, and reliability-regularization objectives. Experience replay is a tool that is only used in training.

MambaAdapt is the complete visual place recognition framework proposed in this study. It consists of the MT-FusionNet descriptor backbone and the CT-MRRE adaptation module, which are jointly optimized using a composite objective function with experience replay. MT-FusionNet integrates convolutional, Mamba state-space, and Transformer components to produce a compact 512-dimensional visual descriptor. CT-MRRE consists of two complementary operations: residual cross-temporal alignment and recurrent context-aware reliability reweighting. During training, experience replay is used to preserve previously learned place representations and mitigate representation drift. During inference, replay memory and training losses are not required; input observations are encoded, adapted to the current context, and then compared with the reference database using cosine similarity. The overall architecture of MambaAdapt is presented in Figure 1, illustrating the distinction between the training and inference phases, the joint optimization of MT-FusionNet and CT-MRRE, and the use of experience replay only during training.

### 3.1. MT-FusionNet Descriptor Backbone

**Figure 2:**
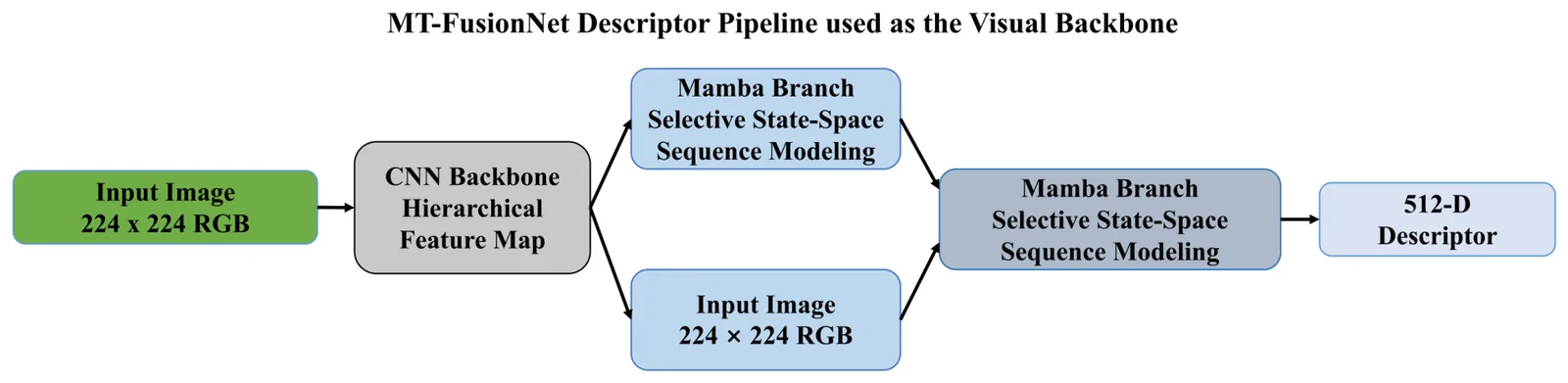
MT-FusionNet descriptor pipeline used as the visual backbone within MambaAdapt.

The MT-FusionNet descriptor backbone in MambaAdapt is illustrated in Figure 2. The complementary Mamba state-space and Transformer attention branches are added to the hierarchical CNN features, concatenated, and projected into a compact 512-D descriptor.

Given an input image $x_{t}$, MT-FusionNet generates a compact visual descriptor by integrating convolutional spatial features, Mamba state-space representations, and Transformer attention features:

(1) $$z_{t}=F\left([C\left(x_{t}\right),M\left(x_{t}\right),T\left(x_{t}\right)]\right)$$

In Equation (1), C(·), M(·), and T(·) denote the CNN feature extractor, Mamba encoder, and Transformer branch; F(·) is the learnable fusion projection; and $z_{t}\in R^{512}$ denotes the final 512-dimensional fused descriptor. The CNN backbone follows the configuration reported in MT-FusionNet [14]. The Mamba branch has a 256-D hidden state, and the Transformer branch is four-layer, with 8 attention heads. They are concatenated and projected first and then aligned and weighted for reliability using CT-MRRE.

### 3.2. Cross-Temporal Alignment Branch Within CT-MRRE

**Figure 3:**
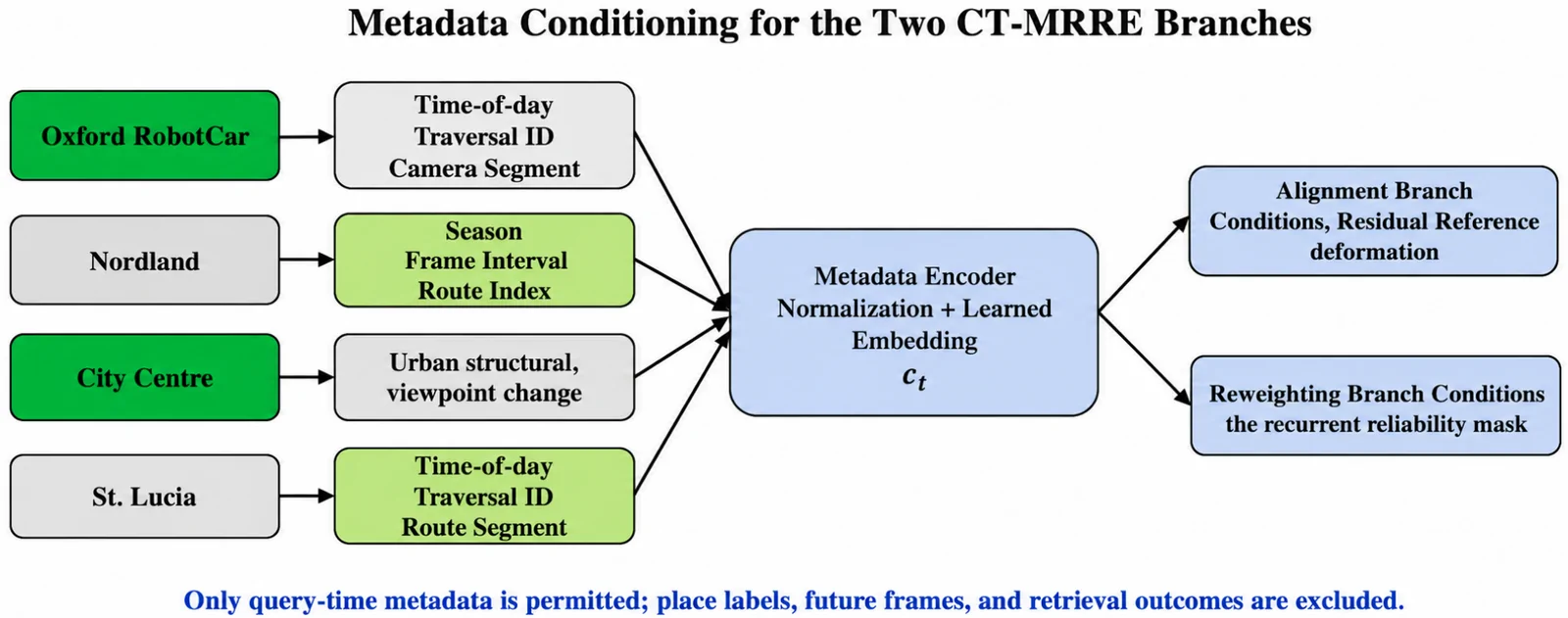
Query-time metadata conditioning for CT-MRRE alignment and recurrent reliability weighting.

Only context available at query time, e.g., time of day, season, traversal identity, route segment, camera segment, and temporal gap, is passed to CT-MRRE. The shared context embedding between the two branches of the alignment and the reliability branches is summarized in Figure 3. Place labels, future observations, and retrieval outcomes are excluded from the context input.

The difference between a reference descriptor $z_{r}$ and a query descriptor $z_{q}$ could be caused by the capture condition, not by the change of place. The alignment branch tries to learn a context-based, nonlinear mapping from the reference representation towards the query condition:

(2) $${\hat{z}}_{r}=z_{r}+T_{\theta }\left(z_{r},\Delta t,c_{t}\right)$$

In Equation (2), $z_{r}$ is the original reference descriptor, ${\hat{z}}_{r}$ is its aligned representation, Δt is the temporal gap, and $c_{t}$ is the query-time context vector. $T_{\theta }:R^{512}\to R^{512}$ denotes the learnable nonlinear residual transformation that maps the original descriptor into a condition-adapted representation. The residual form preserves an identity path while allowing $T_{\theta }$ to learn condition-dependent descriptor deformation. A three-layer ReLU MLP is used to produce the residual deformation from the descriptor, temporal gap, and context embedding. The nonlinear mapping is necessary because road scenes, railway scenes for different seasons, and cluttered urban scenes have different types of drift that cannot be modeled with a single linear transform. The alignment loss is then defined as

(3) $$L_{\mathrm{align}}={∥{\hat{z}}_{r}-z_{q}∥}_{2}^{2}.$$

### 3.3. Recurrent Meta-Reweighting Branch Within CT-MRRE

**Figure 4:**
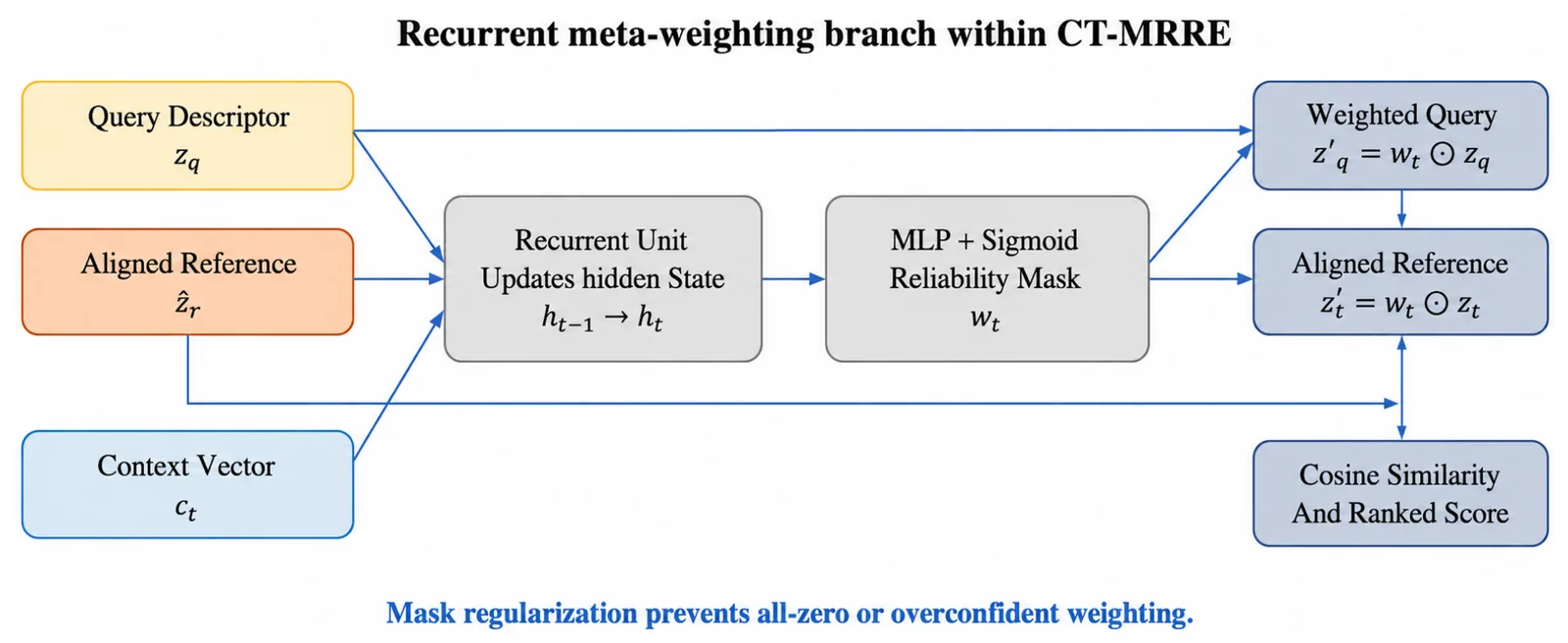
Recurrent reliability weighting within CT-MRRE and context-aware descriptor comparison.

The recurrent meta-reweighting branch is shown in Figure 4. The implementation uses an MLP and sigmoid normalization to update a recurrent hidden state from the previous state, context, query descriptor, and aligned reference descriptor and to predict a 512-D reliability mask.

Descriptor dimensions are not equally effective under different conditions: color descriptors can be informative in the daytime but unreliable in the nighttime; vegetation texture can be informative in summer and unreliable in snow. Here, $w_{t}$ denotes the reliability mask, $h_{t}$ the recurrent hidden state, $c_{t}$ the context vector, σ(·) the sigmoid function, and ⊙ element-wise multiplication:

(4) $$h_{t}=\mathrm{RecurrentUnit}\left(h_{t-1},z_{q},{\hat{z}}_{r},c_{t}\right),$$

(5) $$w_{t}=\sigma \left(\mathrm{MLP}(h_{t},c_{t})\right),$$

(6) $$z_{q}^{w}=w_{t}⊙z_{q},\;z_{r}^{w}=w_{t}⊙{\hat{z}}_{r}.$$

In Equations (4)–(6), $h_{t}$ denotes the recurrent hidden state, $w_{t}\in {\left[0,1\right]}^{512}$ is the context-dependent reliability mask, $c_{t}$ is the query-time context vector, σ(·) is the sigmoid function, and ⊙ denotes element-wise multiplication. The same reliability mask is applied to both the query and the aligned reference descriptors so that unreliable dimensions are suppressed consistently before similarity computation. All subsequent losses (triplet, cross-entropy, stability, and replay) are evaluated on the final reliability-weighted descriptors $z^{w}$, so that the objectives optimize the same representation later used for cosine-similarity ranking.

### 3.4. Experience Replay for Continual Adaptation

During training, a stratified replay buffer, with 1000 temporally diverse samples, is also kept to prevent catastrophic forgetting in the adaptation phase. Samples are stored with stratification by traversal, challenge category, time of day and season to avoid any dominant environmental condition influencing the memory. When buffer fills, replacement is done in the most overrepresented stratum. Redundancy is measured as the highest cosine similarity of a stored sample’s descriptor (512-dimensional) to all the descriptors of observations stored for the same place within the same stratum. The sample that has the highest redundancy score is removed and thus those that are both visually and temporally more diverse are retained.

At each optimization step, current and replay samples are mixed at a 1:1 ratio. Given the effective batch size of 32 in this study, each optimization batch thus consists of 16 current samples and 16 replay samples. Replay consistency stabilizes inaccurate changes of stored representations and lets the model deal with new environmental conditions. In particular, Equation (7) basically promotes low error between the stored replay descriptor and its adapted version. This does not require replay memory, which is attached to an arbitrary model and is only active during training.

(7) $$L_{\mathrm{replay}}={∥z_{\mathrm{new}}-z_{\mathrm{old}}∥}_{2}^{2}.$$

In Equation (7), $z_{\mathrm{old}}$ represents the stored descriptor from the replay memory and $z_{\mathrm{new}}$ represents the updated descriptor after adaptation. Moreover, it constrains the adapted representation of replay samples to remain close to their stored representation, thereby reducing catastrophic forgetting across previously learned conditions.

### 3.5. MambaAdapt Joint Objective Function

**Figure 5:**
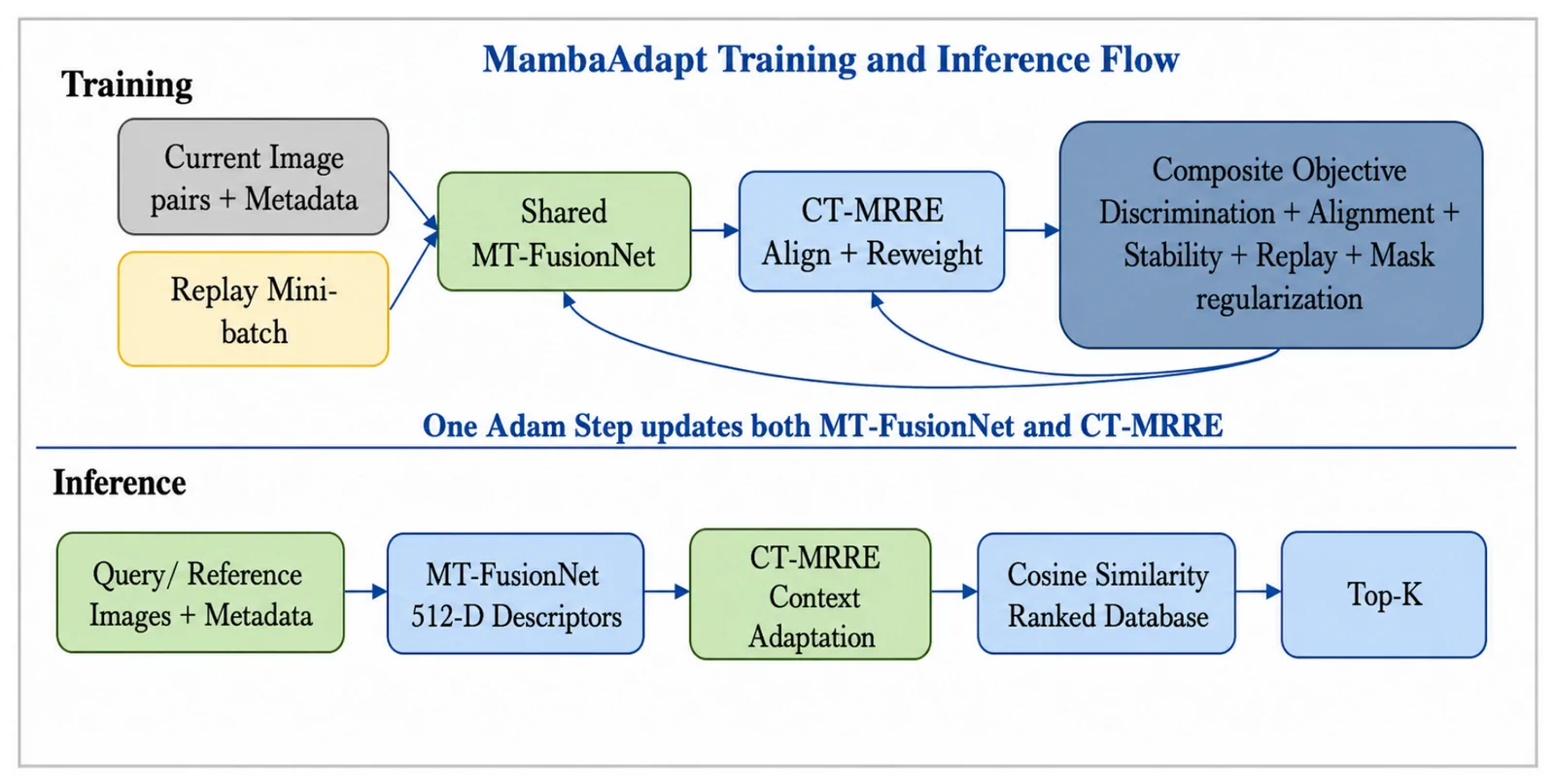
End-to-end MambaAdapt training and inference paths.

In Figure 5, a separation between the end-to-end training path and inference is shown. In training, the current and replay samples go through MT-FusionNet and CT-MRRE, and both modules are updated under the composite objective. During inference, the same MT-FusionNet + CT-MRRE graph is used to generate context-adapted descriptors and ranked cosine-similarity scores; replay memory and training losses are not required.

All components of MambaAdapt are trained jointly with a combined loss that encompasses discrimination, temporal correction, stability, and memory retention:

(8) $$L_{\mathrm{total}}=\lambda_{\mathrm{tri}}L_{\mathrm{tri}}+\lambda_{\mathrm{ce}}L_{\mathrm{ce}}+\lambda_{\mathrm{align}}L_{\mathrm{align}}+\lambda_{\mathrm{stab}}L_{\mathrm{stab}}+\lambda_{\mathrm{replay}}L_{\mathrm{replay}}+\lambda_{\mathrm{reg}}L_{\mathrm{reg}}.$$

The total objective in Equation (8) consists of the triplet discrimination loss $L_{\mathrm{tri}}$, place-classification loss $L_{\mathrm{ce}}$, cross-temporal alignment loss $L_{\mathrm{align}}$, descriptor-stability loss $L_{\mathrm{stab}}$, replay-consistency loss $L_{\mathrm{replay}}$, and reliability-mask regularization loss $L_{\mathrm{reg}}$. Same place pairs for $L_{\mathrm{stab}}$ are drawn from the mini-batch whenever two frames share a place label and are separated by at most one minute; the term is set to zero otherwise. The contribution of each term is controlled by its corresponding weighting coefficient λ. The objective terms and the coefficients are given in Table 1.

The primary retrieval objective remains the triplet loss and is therefore assigned the reference weight $\lambda_{\mathrm{tri}}=1.0$. The remaining coefficients are set relative to this reference so that the auxiliary objectives support representation discrimination without dominating it: cross-entropy classification strengthens place discrimination, alignment and stability losses shape the cross-temporal representation, replay consistency preserves early place associations, and mask regularization prevents degenerate feature weighting. All coefficient values were selected exclusively on the validation traversals by maximizing mean Recall@1; test traversals were never used during hyperparameter selection. A dedicated sensitivity analysis of the principal CT-MRRE and replay-related settings is provided in Section 5.3 and confirms robustness around the chosen operating points. The overall goal is to balance discriminatory power with adaptive robustness.

### 3.6. Training and Inference Procedure

The training and inference procedures are summarized in Algorithm 1. Images and query-time metadata are preprocessed and synchronized, encoded by shared MT-FusionNet weights, aligned and reweighted by CT-MRRE, and combined with a stratified replay mini-batch. At each Adam step, the entire MambaAdapt model is updated jointly.

During inference, the reference database is ranked by cosine similarity. We report Recall@1 as the primary metric and also support Top-K retrieval. The query–reference similarity is defined as

(9) $$S\left(q,r\right)=\frac{{\left(z_{q}^{w}\right)}^{⊤}z_{r}^{w}}{{∥z_{q}^{w}∥}_{2}{∥z_{r}^{w}∥}_{2}},$$

where $z_{q}^{w}$ and $z_{r}^{w}$ denote the query and reference descriptors, respectively.

At inference time, reference descriptors are pre-computed offline using MT-FusionNet and stored in the database. When a query arrives, CT-MRRE computes the reliability mask $w_{t}$ (and optionally the residual alignment) conditioned on the query-time context $c_{t}$ and temporal gap Δt. The same mask $w_{t}$ is applied to both the query descriptor and every reference descriptor (see Equations (6)). This design keeps the database static while still enabling context-aware reweighting at query time. If a temporal gap is available for a given reference, the residual alignment ${\hat{z}}_{r}=z_{r}+T_{\theta }\left(z_{r},\Delta t,c_{t}\right)$ can be applied on-the-fly before masking; otherwise the residual is set to zero (identity mapping).
**Algorithm 1** MambaAdapt—training and inference.**Require:** Image pairs $\left\{I_{q},I_{\mathrm{ref}}\right\}$, metadata, replay buffer B (|B|=1000), initial hidden state $h_{0}=0$
**Training**
 1:**for** epoch =1…50 **do** 2:   **for** each mini-batch $(I_{q},I_{\mathrm{ref}},\mathrm{meta},y)\in D$ **do** 3:      $\left({\mathrm{img}}_{q},{\mathrm{img}}_{\mathrm{ref}},c_{t},\Delta t\right)\leftarrow \mathrm{PreprocessAndSync}\left(I_{q},I_{\mathrm{ref}},\mathrm{meta}\right)$ 4:      $z_{q}\leftarrow \mathrm{MTFusionNet}\left({\mathrm{img}}_{q}\right)$; $z_{\mathrm{ref}}\leftarrow \mathrm{MTFusionNet}\left({\mathrm{img}}_{\mathrm{ref}}\right)$ 5:      $\left({\hat{z}}_{\mathrm{ref}},L_{\mathrm{align}}\right)\leftarrow \mathrm{CTMRRE}.\mathrm{Align}\left(z_{\mathrm{ref}},z_{q},\Delta t,c_{t}\right)$ 6:      $\left(z_{q}^{w},z_{\mathrm{ref}}^{w},w_{t},h_{t},L_{\mathrm{reg}}\right)\leftarrow \mathrm{CTMRRE}.\mathrm{Reweight}\left(z_{q},{\hat{z}}_{\mathrm{ref}},c_{t},h_{t-1}\right)$ 7:      Sample same-place temporal pairs from the current batch and compute $L_{\mathrm{stab}}$ 8:      Compute triplet loss $L_{\mathrm{tri}}$ and cross-entropy loss $L_{\mathrm{ce}}$ on the weighted descriptors $\left\{z^{w}\right\}$ 9:      $\left({\mathrm{batch}}^{\prime },L_{\mathrm{replay}}\right)\leftarrow \mathrm{ExperienceReplay}\left(B,\left\{z_{q}^{w},z_{\mathrm{ref}}^{w}\right\}\right)$10:      $L_{\mathrm{total}}\leftarrow \lambda_{\mathrm{tri}}L_{\mathrm{tri}}+\lambda_{\mathrm{ce}}L_{\mathrm{ce}}+\lambda_{\mathrm{align}}L_{\mathrm{align}}+\lambda_{\mathrm{stab}}L_{\mathrm{stab}}+\lambda_{\mathrm{replay}}L_{\mathrm{replay}}+\lambda_{\mathrm{reg}}L_{\mathrm{reg}}$11:      $\mathrm{Backpropagate}(L_{\mathrm{total}})$; AdamStep(η)12:      $\mathrm{UpdateReplayBuffer}(B,\left\{z_{q}^{w},z_{\mathrm{ref}}^{w}\right\})$       ▹ store weighted descriptors13:   **end for**14:   early-stop on validation Recall@115:**end for**
**Inference**
16:$z_{q}\leftarrow \mathrm{MTFusionNet}\left(I_{q}\right)$17:$\left(z_{q}^{w},w_{t},h_{t}\right)\leftarrow \mathrm{CTMRRE}\left(z_{q},c_{t},\Delta t,h_{t-1}\right)$18: 19:**for** each pre-computed reference descriptor $z_{r}$ in the database **do**20:   ${\hat{z}}_{r}\leftarrow z_{r}+T_{\theta }\left(z_{r},\Delta t,c_{t}\right)$            ▹ residual = 0 if Δt unavailable21:   $z_{r}^{w}\leftarrow w_{t}⊙{\hat{z}}_{r}$22:**end for**23:$\mathrm{scores}\leftarrow \mathrm{CosineSimilarity}(z_{q}^{w},\left\{z_{r}^{w}\right\})$24:return ranked Top-K and Recall@1


## 4. Experimental Setup

### 4.1. Datasets

MambaAdapt was tested on four public datasets: Oxford RobotCar (day–night, weather, traffic, and long-term structural variability) [15]; Nordland (seasonal railway change) [16]; City Centre (urban viewpoint, occlusion, repetitive structure) [17]; and St Lucia (road-based time of day and shadow variation) [17]. City Centre and St Lucia were assessed according to the conventions integrated in VPR-Bench [38]. Table 2 presents the type of challenge and the metadata provided at query time by CT-MRRE.

### 4.2. Dataset Partitioning and Reproducibility Protocol

Training, validation, and test traversals were not allowed to overlap to avoid place-level information leakage. The standard pair traversals for day–night and long-term traversals were used for evaluation, and the training and validation traversals were sampled from different traversals. In Nordland, the sequences for seasonal reference and query were split based on the benchmark protocol, with the aim of keeping a significant seasonal variation during evaluation. The evaluation of City Centre and St Lucia was based on conventions that are part of VPR-Bench. Furthermore, positive pairs were query and reference images taken from the same physical location at different times of the day, in different conditions. The negatives were chosen from similar visual regions but different geographic regions (hard negatives) and did not include trivially easy (easy, but not hard) negatives. The results presented in the current study are based on the average of five independent runs (with different random seeds) of the trained models. Non-parametric bootstrap resampling (with 2000 resamples of the query-level correctness indicators) was used to compute confidence intervals.

### 4.3. Implementation Details

Table 3 summarizes the implementation. Images were resized to 224×224 and normalized according to the ImageNet statistics. The MT-FusionNet descriptor configuration followed our previous implementation [14] and used an ImageNet-pretrained ResNet-50 backbone for hierarchical spatial feature extraction, a 256-dimensional Mamba state, a four-layer Transformer with eight attention heads, and a fused 512-dimensional descriptor. CT-MRRE used a three-layer ReLU alignment MLP and a 256-D recurrent hidden state. The replay buffer contained 1000 samples. Adam was initialized at 0.001, then gradually decreased by 0.1 every 10 epochs, with a batch size of 32, a maximum of 50 epochs, and early stopping after five validation epochs with no improvement. Training was performed on the GTX 1060 setup of [32] using mixed-precision training, and latency was also profiled on a modern desktop reference, the RTX 4090. Separate profiling must be performed for each hardware platform with embedded deployment.

### 4.4. Evaluation Protocol

The percentage of query images for which the correct reference image was retrieved at the first rank, Recall at rank one, Recall@1, was used as the primary evaluation measure. The average Recall@1 of all four evaluation datasets was used to summarize overall performance. In addition, the results presented in repeated experiments in this study are the mean over five separate experiments with different random seed values. They use non-parametric bootstrap resampling with 2000 resamples of the correctness indicators at the query level to calculate their 95% confidence intervals. The results for CerfeVPR and MT-FusionNet are taken from our previous study on unified evaluation, where we evaluated on Oxford RobotCar, Nordland, City Centre, and St Lucia. These previous-work results are reported as point estimates only and are indicated in the comparison table with a dagger symbol because the seed-level confidence intervals were not reported. NetVLAD [2], CosPlace [3], MixVPR [4], EigenPlaces [5], CerfeVPR [38], MT-FusionNet [14], and the proposed MambaAdapt framework were the evaluated methods. CT-MRRE was not evaluated as a standalone VPR model, as it functions as an adaptation module within MambaAdapt and operates on descriptors produced by the MT-FusionNet backbone.

## 5. Results and Discussion

### 5.1. Overall Comparison with Baselines

**Figure 6:**
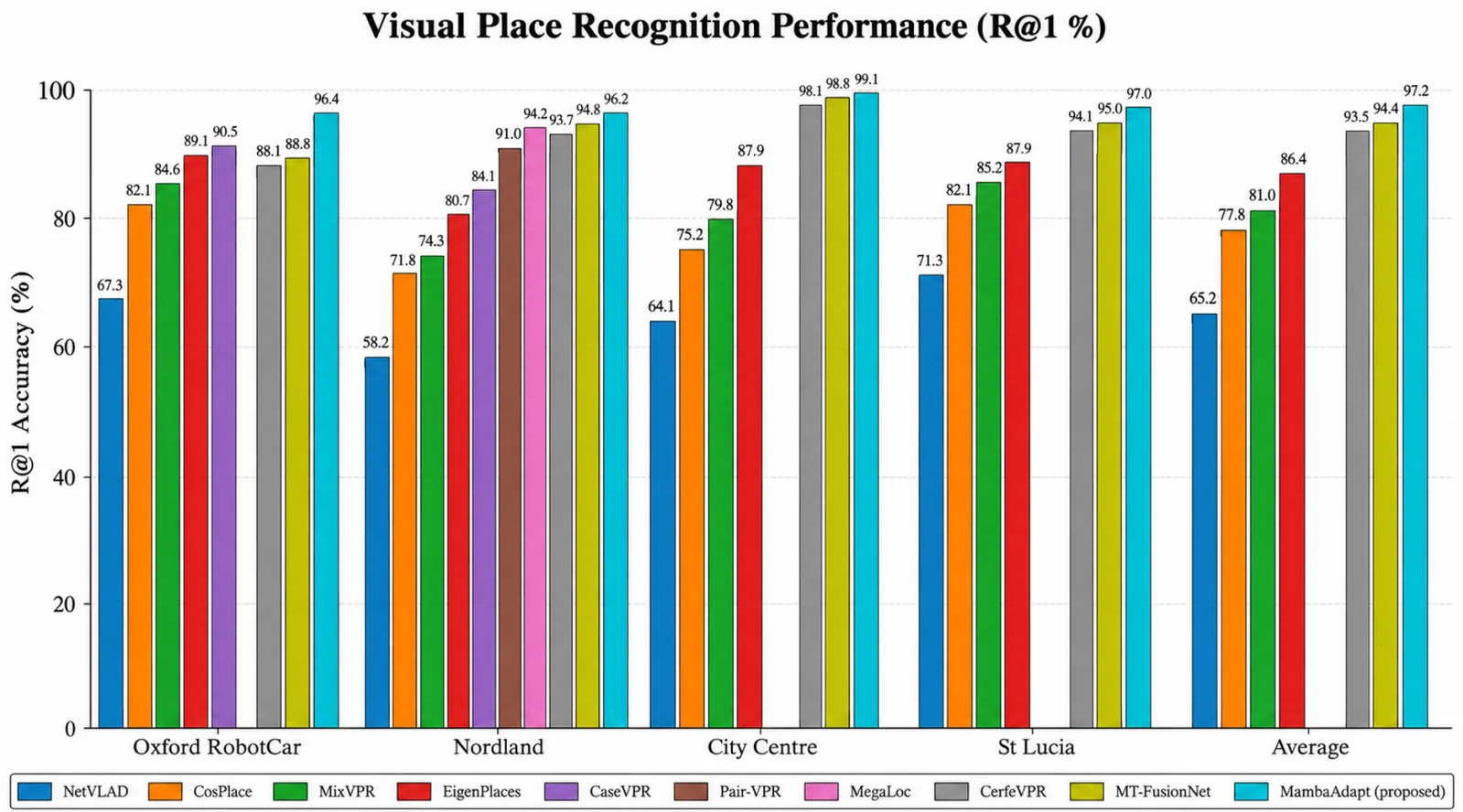
Recall@1 comparison across Oxford RobotCar, Nordland, City Centre, and St Lucia. CerfeVPR and MT-FusionNet values are point estimates from our previous unified evaluation, while MambaAdapt results are reported as the mean over five independent runs.

In the common four-datasets protocol, MambaAdapt outperformed all the other methods according to average Recall@1 with 97.2% compared to 94.4% by MT-FusionNet, 93.5% by CerfeVPR, 86.4% by EigenPlaces, 81.0% by MixVPR, 77.8% by CosPlace, and 65.2% by NetVLAD. The Protocol-Meta (CaseVPR, Pair-VPR and MegaLoc) results in Table 4 are literature-reported reference results that do not correspond to the present protocol and are therefore to be evaluated individually and not as protocol-matched comparisons. We compared MambaAdapt against well-known global-descriptor approaches, the recent CerfeVPR approach and our previously introduced MT-FusionNet model in Table 4 and Figure 6. MambaAdapt achieved 96.4%, 96.2%, 99.1%, and 97.0% Recall@1 on Oxford RobotCar, Nordland, City Centre, and St Lucia, respectively, with an average Recall@1 of 97.2%. Furthermore, MambaAdapt outperformed CerfeVPR by 8.3 percentage points on Oxford RobotCar, 2.5 points on Nordland, 1.0 point on City Centre, and 2.9 points on St Lucia.

The average gain over CerfeVPR was 3.7 percentage points. MambaAdapt outperformed MT-FusionNet by 7.6 points on Oxford RobotCar, 1.4 points on Nordland, 0.3 points on City Centre, and 2.0 points on St Lucia, which is an average of 2.8 points across the four datasets. On Oxford RobotCar, the large variations in visual appearance (daytime/nighttime, shadow/glare, weather and traffic) are particularly challenging. The cross-temporal alignment branch corrected for the descriptor drift caused by these condition changes, and reliability reweighting helped reduce the influence of features that change considerably under illumination variation. On Nordland and St Lucia, the improvements indicated higher robustness to seasonal and time-of-day changes. The smaller improvement on City Centre was expected because CerfeVPR and MT-FusionNet already reached high values of 98.1% and 98.8%, respectively, leaving limited room for further gains. Overall, these results demonstrate the effectiveness of the proposed joint descriptor–adaptation strategy under diverse long-term visual conditions. Note that the CerfeVPR and MT-FusionNet values are point estimates from previous work and should be viewed descriptively rather than as repeated experiments in the present study.

### 5.2. Incremental Component Analysis

Table 5 presents the progressive addition of the main components used in MambaAdapt. The MT-FusionNet row is the previous reported performance of the descriptor-backbone, whereas the other rows show the impact of cross-temporal alignment, recurrent reliability reweighting, and experience replay in the current setting.

Since the MT-FusionNet row is a point estimate from previous work and not the average of successive point estimates from the current study, this analysis is treated as an incremental comparison to the component, rather than a statistically controlled ablation. Moreover, the cross-temporal alignment results in the most significant gain on Oxford RobotCar, suggesting that explicit correction of descriptor drift is especially crucial under day–night variation. Recurrent reliability reweighting further improves performance by suppressing dimensions which are unreliable during varying illumination, vegetation, shadow, viewpoints, or occlusion. Experience replay can offer an extra benefit of retaining previously learned place representations. The full MambaAdapt setup performs best on all four datasets in terms of Recall@1.

**Figure 7:**
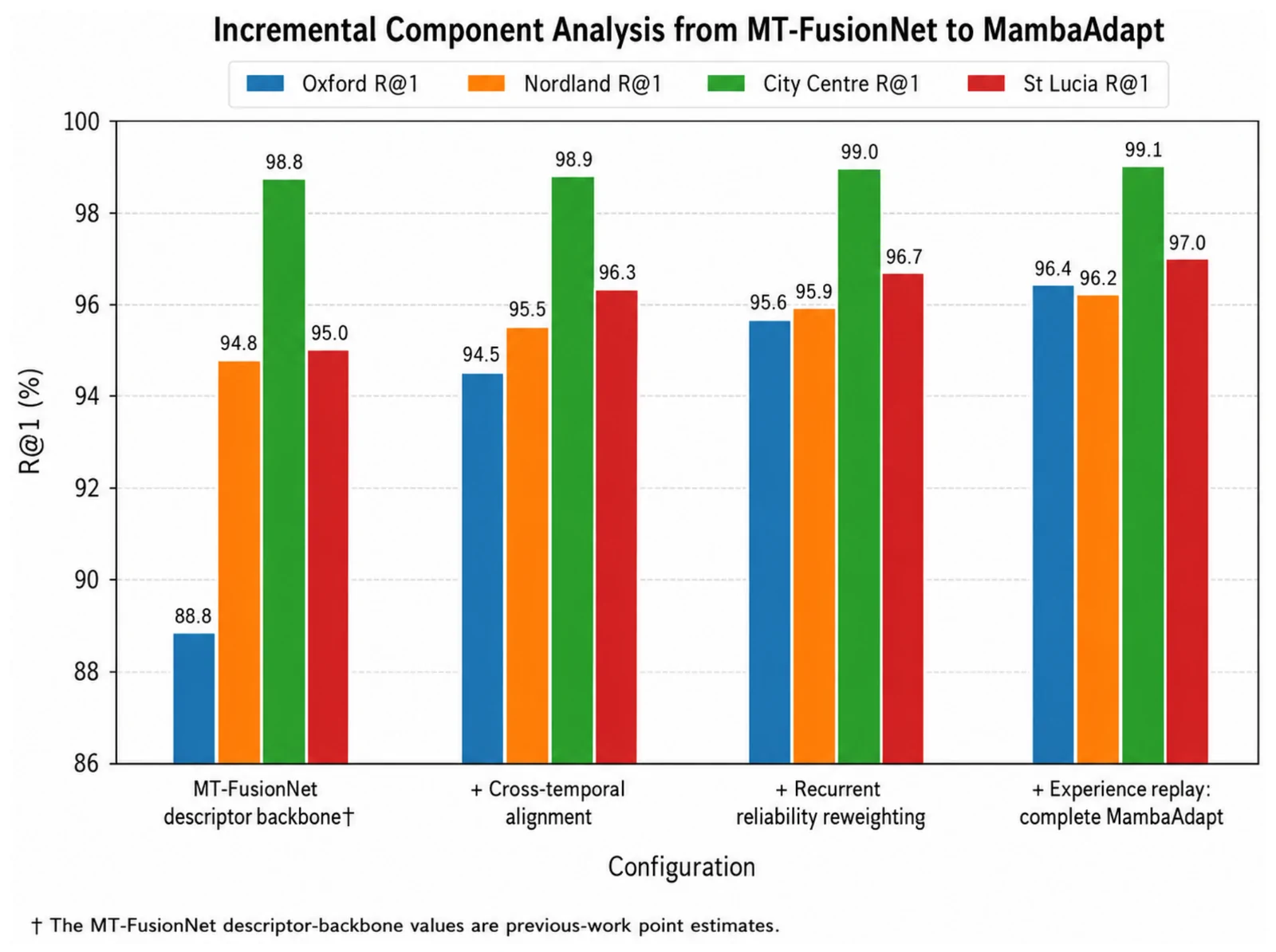
Incremental ablation from MT-FusionNet to full MambaAdapt. Each stage adds one component: cross-temporal alignment, complete CT-MRRE (alignment + recurrent reliability reweighting), and experience replay.

Figure 7 presents the same incremental results in graphical form. The most noticeable improvement appears after adding the cross-temporal alignment branch, particularly on the Oxford RobotCar dataset. Subsequent addition of recurrent reliability reweighting and experience replay brings further consistent gains. The complete MambaAdapt model obtains the best Recall@1 on every dataset.

### 5.3. Parameter Sensitivity Analysis

To examine the sensitivity of MambaAdapt to its principal hyperparameters, a one-factor-at-a-time analysis was conducted using the validation traversals, while all other training settings were kept fixed. The test traversals were not used during parameter selection. As summarized in Table 6, the analysis considered the cross-temporal alignment-loss weight ($\lambda_{\mathrm{align}}$), replay-consistency weight ($\lambda_{\mathrm{replay}}$), reliability-regularization weight ($\lambda_{\mathrm{reg}}$), replay-buffer capacity, and CT-MRRE recurrent hidden-state dimension. The tested values were centered around the configuration used in the final model: ($\lambda_{\mathrm{align}}=0.70$), ($\lambda_{\mathrm{replay}}=0.40$), ($\lambda_{\mathrm{reg}}=0.10$), a replay-buffer size of 1000 samples, and a recurrent hidden-state dimension of 256.

The sensitivity analysis provides empirical justification for the selected configuration rather than treating these parameters as arbitrary design choices. The alignment coefficient controls the contribution of cross-temporal descriptor correction, whereas the replay-consistency coefficient balances adaptation to current conditions with retention of previously learned place representations. The reliability-regularization coefficient constrains excessively sparse or overconfident feature masks. Similarly, replay-buffer capacity determines the diversity of historical conditions retained during training, while the recurrent hidden-state dimension controls the representational capacity of the reliability-reweighting mechanism. Based on the validation Recall@1 values reported in Table 6, the selected settings provided the best balance among retrieval accuracy, temporal adaptation, representation retention, and model complexity and were therefore retained unchanged for the final test evaluation.

### 5.4. Post Hoc MT-FusionNet + CT-MRRE Versus End-to-End MambaAdapt

In Table 7, we compare two training methods while using the same MT-FusionNet + CT-MRRE inference architecture. In Condition A, the MT-FusionNet backbone was frozen, and only CT-MRRE was trained. In Condition B, the entire MambaAdapt model was trained end-to-end, enabling co-adaptation of the objectives in the descriptor, alignment, reliability, stability, and replay. The number of data partitions, batch size, and the learning-rate schedule were fixed, as were the capacity of the replay memory and the sampling and optimization budget.

The end-to-end MambaAdapt achieves better results in all benchmarks: +1.2 points in Oxford RobotCar, +1.4 points in Nordland, +3.0 points in City Centre, and +1.3 points in St Lucia. Average Recall@1 rises from 95.45% to 97.18%, a 1.73-point absolute gain. This comparison focuses on the benefit of joint representation learning instead of other deployment modules, as the inference graph remained unchanged.

### 5.5. Environmental Robustness

MambaAdapt always outperformed MT-FusionNet and CerfeVPR in the four main environmental conditions covered by the four evaluation datasets. The best performance gain was found on Oxford RobotCar, with MambaAdapt achieving a 7.6% and 8.3% improvement in Recall@1 over MT-FusionNet and CerfeVPR, respectively, under extreme day-to-night illumination variation. The system MambaAdapt outperformed MT-FusionNet and CerfeVPR by 1.4 and 2.5 points, respectively, on Nordland, which shows enhanced adaptability against snow coverage, vegetation change and seasonal color variation.

Under viewpoint variation, dynamic vehicle occlusion, and repetitive urban structure, MambaAdapt achieved 99.1% Recall@1 on City Centre. While the absolute improvement was smaller, the result was still higher than the already high MT-FusionNet and CerfeVPR results. MambaAdapt outperformed MT-FusionNet by 2.0 points and CerfeVPR by 2.9 points in the presence of varying sun angles, shadows, and appearance variation across different times of day, on St Lucia.

These consistent improvements across different types of appearance variation support the effectiveness of the joint descriptor–adaptation strategy under the tested conditions. However, the results remain limited to the four evaluated datasets; generalization to unseen locations, cameras, and motion patterns remains an important direction for future work.

### 5.6. Computational Efficiency and Deployment Feasibility

Table 8 shows the computational properties of MambaAdapt compared to representative VPR systems in legacy and modern desktop GPUs architectures. MambaAdapt adds a moderate amount of computation: it scales by 39.6 GFLOPs and involves 124.8 million parameters, mainly from the added CT-MRRE adaptation module, which performs cross-temporal alignment and reliability-aware feature reweighting. When comparing MambaAdapt with its backbone model MT-FusionNet, the former has about 11% more parameters and 2.4/0.5 ms/query more latency on GTX 1060/RTX 4090 respectively, which shows that the adaptation mechanism has only a small overhead compared to the increased robustness. While lightweight methods like NetVLAD, CosPlace, MixVPR, and EigenPlaces have lower computational costs, they do not have explicit mechanisms for dealing with long-term environmental variations. MambaAdapt 512-dimensional descriptor guarantees compact retrieval representation yet keeps its discriminative capacity. The results overall show that MambaAdapt is a viable approach for real-time VPR systems on desktops but can be further optimized by pruning, distillation and quantization for deployment on resource limited platforms.

### 5.7. Qualitative Error Analysis

To better understand the remaining errors, we inspected both successful and failed top-one retrievals. For each case we noted the type of appearance or viewpoint change in the query, which structural elements stayed consistent across traversals, which nuisance features CT-MRRE down-weighted, and whether the correct place was retrieved. Successes were queries where the right reference still ranked first despite large visual change. Failures were rank-one mismatches in which the useful place evidence was missing, occluded, or simply not distinctive enough. These residual mistakes are different from ordinary condition drift, where the structure is still present but temporarily overwhelmed by appearance variation.

On Oxford RobotCar the method often kept building façades, lane markings, road edges and tree lines even under strong day-to-night and shadow/glare changes; reliability reweighting helped suppress the brightness-sensitive cues. Nordland successes usually relied on railway tracks, bridges and the horizon line when vegetation and snow made color unreliable. In City Centre, correct matches tended to preserve road layout and façade geometry despite lateral viewpoint shifts or partial vehicle occlusion. St Lucia cases frequently matched on road curvature, poles, skyline and tree lines despite changing sun angle and saturation. Most residual failures happened when these stable cues were either destroyed, heavily blocked, or repeated in several nearby locations.

**Figure 8:**
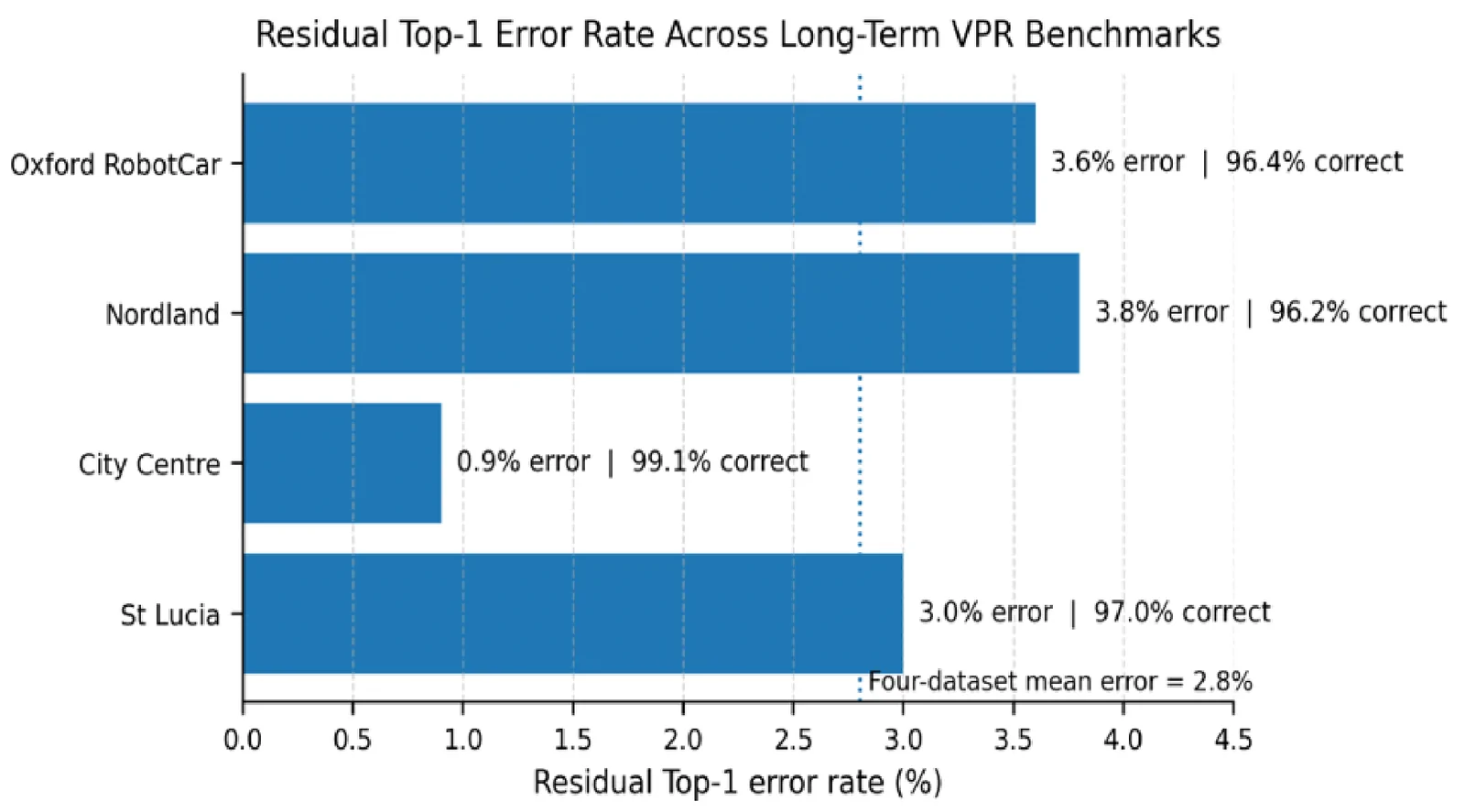
Residual top-1 error rate across the four benchmarks, calculated as 100 − Recall@1. The labels also report the corresponding successful top-1 retrieval rate.

Figure 8 shows the residual top-one error (100−Recall@1) on the four benchmarks: 3.6% on Oxford RobotCar, 3.8% on Nordland, 0.9% on City Centre and 3.0% on St Lucia (mean 2.8%). Nordland remains the hardest, largely because of dense snow and long stretches of visually similar railway. City Centre is almost solved. These numbers describe overall dataset performance and do not tell us how often each individual failure mode occurs.

**Figure 9:**
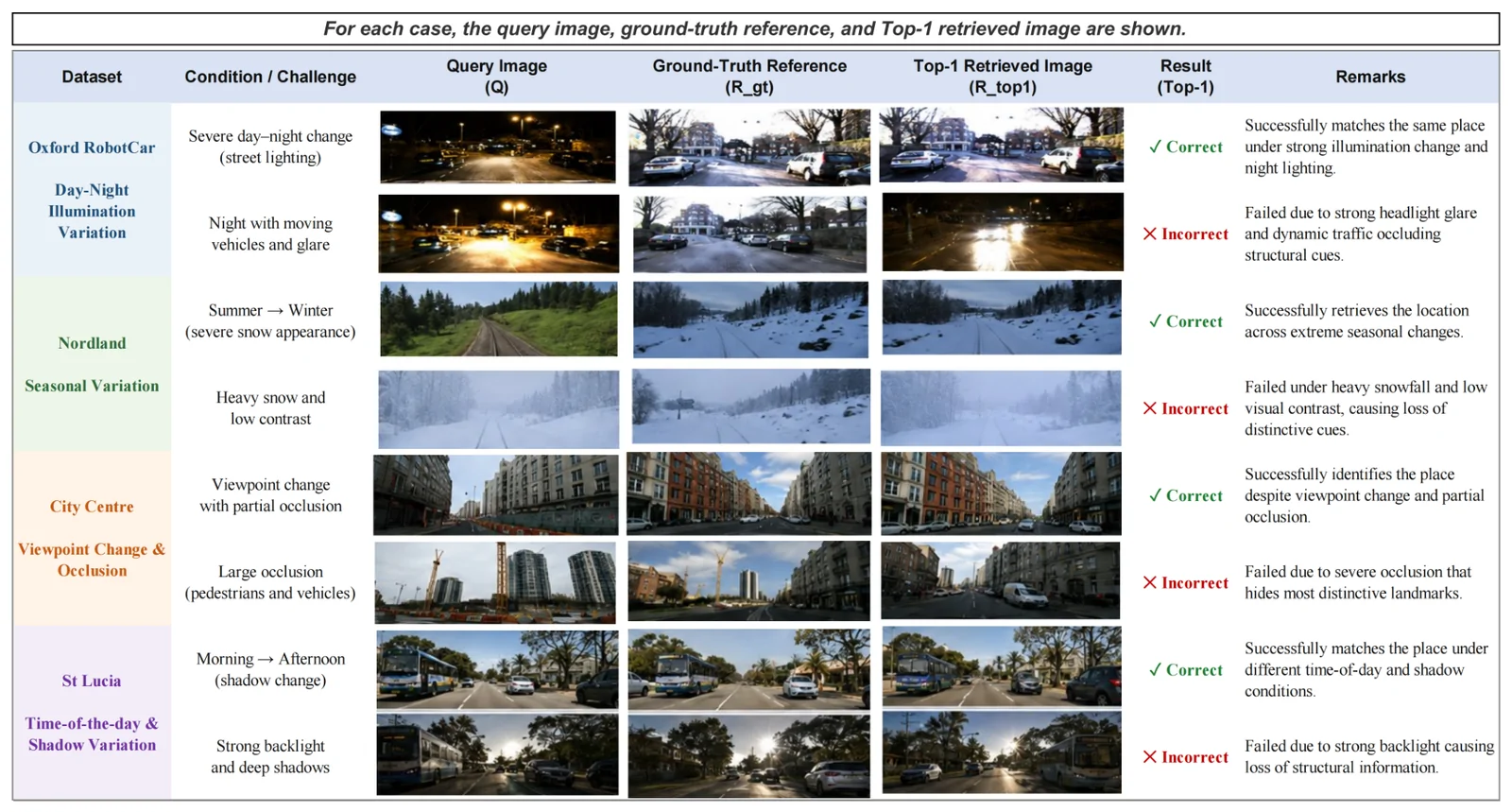
Representative successful and failed top-1 retrieval cases of MambaAdapt across the four evaluated benchmarks. For each case, the query image, ground-truth reference, and top-1 retrieved image are shown.

In addition, Figure 9 shows representative retrieval outputs so that qualitative observations can be verified at a glance. For each benchmark a query image, its ground-truth reference, and the top-one result returned by MambaAdapt are presented, covering both successful and failed cases. The examples illustrate extreme day/night illumination differences, seasonal appearance changes, viewpoint variation with occlusion, and time-of-day/shadow changes. Successful retrievals retain sufficient distinctive structural information to compensate for appearance variation, whereas failures typically occur under strong occlusion, deep snow, repetitive geometry, or loss of locally identifying visual structure.

In short, MambaAdapt works well when a place changes appearance but still keeps a recognizable structural signature. The remaining errors mainly appear when that structure is absent, fully occluded, or highly ambiguous. This points toward future work on uncertainty-aware retrieval, better detection of perceptual aliases, sequence-level checks, and simple fallback strategies for the cases where CT-MRRE is not confident.

## 6. Limitations and Future Work

Several limitations remain. The first one is contextual metadata at query time, which is the feature of CT-MRRE. Cross-temporal alignment and reliability estimation might not be as effective due to incomplete, inaccurate, or missing metadata. Future research should thus focus on metadata dropout, uncertainty-aware conditioning and metadata-free fallback operation. Second, the current evaluation is based on the evaluation of adaptation in individual datasets. Generalization of cross-dataset to unseen locations, cameras, routes and motion patterns has not yet been demonstrated. Thirdly, the 124.8-million-parameter MambaAdapt model is resource-intensive, which has inspired studies on model compression, distillation, pruning, quantization, and lightweight backbones. Fourth, the replay mechanism has not been tested under prolonged (repeated environmental) deployments. Lastly, the problem of severe occlusion, repetitive structures, and dense snow are challenging as they either mask or destroy the stable geometric information that is crucial for place matching.

## 7. Conclusions

We proposed a new framework for robust long-term visual place recognition: MambaAdapt. The framework builds upon the MT-FusionNet CNNs–Mamba–Transformer (CNN-Mamba-Transformer) with the proposed Cross-Temporal alignment and Recurrent Context-aware reliability reweighting (CT-MRRE) module. Joint training is completed with experience replay to limit the representation drift and retain place association from the past. The novelty therefore does not only consist of the fusion of the architecture but of an elegant joint optimization of descriptor discrimination, temporal adaptation, context-dependent reliability, and representation preservation.

MambaAdapt achieved Recall@1 scores of 96.4%, 96.2%, 99.1%, and 97.0% on Oxford RobotCar, Nordland, City Centre, and St Lucia, respectively, with an average of 97.2%. This corresponded to average improvements of 2.8 percentage points over MT-FusionNet and 3.7 percentage points over CerfeVPR under the common four-dataset protocol. The largest gain was observed under severe day–night illumination changes on Oxford RobotCar, while consistent improvements were also obtained under seasonal variation, urban viewpoint changes, occlusion, and time-of-day appearance shifts.

Ablation studies showed that cross-temporal alignment, reliability reweighting, and experience replay complemented one another. Promising directions for future work include cross-dataset generalization, robustness to missing contextual metadata, longer continual deployments, and more efficient implementations.

## Figures and Tables

**Table 1:** Composite loss terms, weighting coefficients, and functional roles in MambaAdapt objective.

Loss Term	Expression/Definition	Loss Coefficient/λ Value	Function in MambaAdapt
Triplet Loss	$L_{\mathrm{tri}}=\max\left(0,d\left(z_{a},z_{p}\right)-d\left(z_{a},z_{n}\right)+m\right)$	1.00	Improves place-level descriptor discrimination.
Cross-Entropy Loss	$L_{\mathrm{ce}}=-\sum y\log\left(p\right)$	0.50	Supports supervised place identity learning.
Alignment Loss	$L_{\mathrm{align}}={∥{\hat{z}}_{r}-z_{q}∥}_{2}^{2}$	0.70	Optimizes the residual cross-temporal alignment.
Stability Loss	$L_{\mathrm{stab}}={∥z_{t}^{w}-z_{t-1}^{w}∥}_{2}^{2}$ (same place)	0.30	Encourages temporal consistency of same-place weighted descriptors
Replay Consistency Loss	$L_{\mathrm{replay}}={∥z_{\mathrm{new}}-z_{\mathrm{old}}∥}_{2}^{2}$	0.40	Prevents catastrophic forgetting across previous conditions.
Weight Regularization	$L_{\mathrm{reg}}={∥w_{t}∥}_{2}+H\left(w_{t}\right)$	0.10	Prevents unstable or overconfident reliability masks.

**Table 2:** Benchmark datasets and associated metadata used for temporal conditioning.

Dataset	Primary Setting	Main Challenge	Metadata Used
Oxford RobotCar	Day–night urban traversal	Illumination, weather, dynamic traffic	Time-of-day, traversal ID, camera segment
Nordland	Seasonal railway traversal	Winter/summer shift, snow, vegetation	Season, frame interval, route index
City Centre	Urban street sequence	Viewpoint, occlusion, repetitive structure	Traversal ID, route segment, frame index
St Lucia	Road-based time-of-day traversal	Sun angle, shadow, road appearance	Time-of-day, traversal ID, route segment

**Table 3:** Hyperparameters, configuration, and reproducibility settings of MambaAdapt.

Component	Configuration	Purpose
Input resolution	224×224 RGB	Matches MT-FusionNet’s training protocol
CNN backbone	ResNet-50	Local/mid-level spatial features
Mamba state dim.	256	Selective state-space sequence modeling
Transformer	4 layers, 8 heads	Long-range spatial relationships
Descriptor dim.	512-D	Compact global representation
CT-MRRE alignment MLP	3 hidden layers, ReLU	Learns temporal deformation field
CT-MRRE recurrent hidden size	256	Recurrent context accumulation
Replay buffer	1000 samples	Anti-catastrophic forgetting memory
Optimizer/LR	Adam, 0.001 (×0.1/10 epochs)	Stable joint optimization
Batch/epochs	32/50 (early stopping, patience 5)	Training regime
Seeds/CI	5 seeds; 95% bootstrap CI (2000 resamples)	Statistical reliability of reported metrics

**Table 4:** Recall@1 (%) on four long-term VPR benchmarks. The results whose confidence intervals are shown are the mean ± 95% bootstrap confidence interval over five independent runs. Results for CerfeVPR and MT-FusionNet marked with † are point estimates from our previous unified four-dataset evaluation.

Model	Oxford RobotCar R@1 (%)	Nordland R@1 (%)	City Centre R@1 (%)	St Lucia R@1 (%)	Average R@1 (%)
NetVLAD [2]	67.3±0.6	58.2±0.7	64.1±0.6	71.3±0.5	65.2
CosPlace [3]	82.1±0.5	71.8±0.6	75.2±0.5	82.1±0.4	77.8
MixVPR [4]	84.6±0.5	74.3±0.6	79.8±0.5	85.2±0.4	81.0
EigenPlaces [5]	89.1±0.4	80.7±0.5	87.9±0.4	87.9±0.4	86.4
CaseVPR [10] ‡	90.5/72.8 *	84.1	—	—	—
Pair-VPR [11] ‡	—	91.0	—	—	—
MegaLoc [39] ‡	—	94.2	—	—	—
CerfeVPR † [38]	88.1	93.7	98.1	94.1	93.5
MT-FusionNet † [14]	88.8	94.8	98.8	95.0	94.4
**MambaAdapt (proposed)**	96.4±0.3	96.2±0.4	99.1±0.2	97.2±0.3	97.2

Note: † Point estimates obtained from our previous unified four-dataset evaluation. ‡ Literature-reported result from the cited study under its original evaluation protocol and therefore not directly comparable with the unified four-dataset evaluation used for MambaAdapt. * Result reported as two values under different protocols in the original paper. — indicates that a sufficiently protocol-matched result was not reported and was therefore not imputed.

**Table 5:** Incremental component analysis from the previously reported MT-FusionNet descriptor baseline to the complete MambaAdapt framework.

Configuration	Oxford R@1	Nordland R@1	City Centre R@1	St Lucia R@1
MT-FusionNet descriptor backbone †	88.8	94.8	98.8	95.0
+Cross-temporal alignment	94.5	95.5	98.9	96.3
+Recurrent reliability reweighting	95.6	95.9	99.0	96.7
+Experience replay: complete MambaAdapt	96.4	96.2	99.1	97.0

† The MT-FusionNet descriptor-backbone values are previous-work point estimates.

**Table 6:** Sensitivity analysis of the principal MambaAdapt hyperparameters using validation Recall@1. Bold values indicate the configuration selected for the final model.

Parameter	Values to Test	Selected Value	Validation R@1 (%)
$\lambda_{\mathrm{align}}$	0.5, 0.7, 0.9	0.7	96.55/**97.02**/96.71
$\lambda_{\mathrm{replay}}$	0.2, 0.4, 0.6	0.4	96.48/**96.79**/96.66
$\lambda_{\mathrm{reg}}$	0.05, 0.10, 0.20	0.1	96.73/**97.12**/96.58
Replay buffer size	500, 1000, 2000	1000	96.54/**97.23**/96.91
CT-MRRE hidden size	128, 256, 512	256	96.51/**96.85**/96.84

**Table 7:** Post hoc frozen-backbone versus end-to-end joint training of MambaAdapt. Both configurations use the identical MT-FusionNet + CT-MRRE inference architecture.

Training Regime	Oxford R@1	Nordland R@1	City Centre R@1	St Lucia R@1	Average R@1
(A) Frozen MT-FusionNet + CT-MRRE (post hoc)	95.2±0.3	94.8±0.3	96.1±0.3	95.7±0.3	95.45
(B) MambaAdapt (joint end-to-end)	96.4±0.3	96.2±0.4	99.1±0.2	97.0±0.3	97.18
Δ (B − A)	+1.2	+1.4	+3.0	+1.3	+1.73

**Table 8:** Configuration-specific computational comparison on legacy and modern desktop GPUs.

Model	Parameters (M)	GTX 1060 (ms/Query)	RTX 4090 (ms/Query)	FLOPs (G)	Descriptor Dimension
NetVLAD [2]	25.3	7.4	2.1	15.2	4096
CosPlace [3]	27.4	7.8	2.2	17.8	512
MixVPR [4]	22.6	8.0	2.2	20.4	512
EigenPlaces [5]	24.0	7.9	2.2	18.6	512
CerfeVPR [38]	107.3	8.2	–	–	–
MT-FusionNet [14]	112.5	8.5	2.6	36.8	512
**MambaAdapt**	124.8	10.9	3.1	39.6	512

## Data Availability

All the datasets for the benchmarks used in this study are readily obtainable from their original sources. In the manuscript, the reader can find details about the implementation of these experiments, the model configuration, hyperparameters, protocol for partitioning the dataset, and the evaluation procedure required for reproducing the experiments. The corresponding author will make additional implementation materials, such as configuration files and evaluation information, available on request.

## Abbreviations

The following abbreviations are used in this manuscript:

VPR	Visual Place Recognition
MT-FusionNet	Mamba–Transformer Fusion Network
CT-MRRE	Cross-Temporal Meta-Reweighting Recurrent Encoder
SSM	Selective State-Space Model
CNN	Convolutional Neural Network
MLP	Multilayer Perceptron
ReLU	Rectified Linear Unit
ViT	Vision Transformer
VLAD	Vector of Locally Aggregated Descriptors
RGB	Red, Green, and Blue
GPS	Global Positioning System
GPU	Graphics Processing Unit
FLOPs	Floating-Point Operations
R@1	Recall at Rank 1
CI	Confidence Interval
LR	Learning Rate
Δt	Temporal Gap
